# Identification of novel variations of oculocutaneous albinism type 2 with Prader–Willi syndrome/Angelman syndrome in two Chinese families

**DOI:** 10.3389/fgene.2023.1135698

**Published:** 2023-03-06

**Authors:** XiaoFei Chen, ZiShui Fang, Ting Pang, DongZhi Li, Jie Lei, WeiYing Jiang, HongYi Li

**Affiliations:** ^1^ Maternity and Child Care Center of Dezhou, DeZhou, China; ^2^ Department of Medical Genetics, Zhongshan School of Medicine, Guangzhou, China; ^3^ Beijing Key Laboratory of Urogenital Diseases (Male) Molecular Diagnosis and Treatment Center, National Urological Cancer Center, Department of Urology, Peking University First Hospital, Institution of Urology, Peking University, Beijing, China; ^4^ Prenatal Diagnosis Center, Guangzhou Women and Children’s Medical Center, Guangzhou, China; ^5^ Shenzhen Nanshan Maternity and Child Healthcare Hospital, Shenzhen, China

**Keywords:** Prader–Willi syndrome, Angelman syndrome, oculocutaneous albinism type 2, mutation, prenatal diagnosis

## Abstract

**Objective:** Oculocutaneous albinism (OCA) is an autosomal recessive disorder caused by a variety of genomic variations. Our aim is to identify the molecular basis of OCA in two families and lay the foundation for prenatal diagnosis.

**Methods:** Four types of OCA-causing mutations in the TYR, *p*, TYRP1, or SLC45A2 genes were screened. Linkage analysis was performed because the mutations found in the *p* gene violated the laws of classical Mendelian heredity. Primer-walking sequencing combined with microsatellite and single-nucleotide polymorphism analysis was used to ascertain deletion ranges. Bioinformatics methods were used to assess the pathogenicity of the new mutations.

**Results:** Proband 1 was diagnosed as OCA2 with Prader–Willi syndrome (PWS) due to a novel atypical paternal deletion (chromosome 15: 22330347–26089649) and a pathogenic mutation, c.1327G>A (Val443Ile), in the *p* gene of the maternal chromosome. The prenatal diagnosis results for family 1 indicated the fetus was a heterozygous carrier (c.1327G>A in the *p* gene) with a normal phenotype. Proband 2 was diagnosed as OCA2 with Angelman syndrome (AS) due to a typical maternal deletion of chromosome 15q11-q13 and a novel mutation, c.1514T>C (Phe505Ser), in the *p* gene of the paternal chromosome. This novel mutation c.1514T>C (Phe505Ser) in the *p* gene was predicted as a pathogenic mutation.

**Conclusion:** Our study has shown clear genotype–phenotype correlations in patients affected by distinct deletions of the PWS or AS region and missense mutations in the *p* gene. Our results have enriched the mutation spectrum of albinism diseases and provided insights for more accurate diagnosis and genetic counseling.

## Introduction

Oculocutaneous albinism (OCA), which is characterized by impaired eye development plus variable hair, skin, and ocular hypopigmentation, is a genetically inherited autosomal recessive condition, with a prevalence as high as 1/2000 and a carrier ratio of 1/70 worldwide (Gargiulo et al., 2011). Individuals with OCA are susceptible to the harmful effects of solar ultraviolet radiation, including extreme Sun sensitivity, photophobia, and skin cancer (Wright et al., 2015). Four known genes, *TYR* (OCA1), *p* (OCA2), TYRP1 (OCA3), and SLC45A2 (OCA4) have been isolated in association with OCA. Mutations in these genes affected the correct sorting and trafficking of their respective proteins, which ultimately hampers the maturation of melanosomes and melanin production (Bailus and Segal, 2014; Costin et al., 2003; Kamaraj et al., 2016). Recently, two new genes SLC24A5 and C10orf11 have been identified. The gene SLC24A5 (OCA5), is involved in the maturation of melanosomes, whereas gene C10orf11 (OCA6) is involved in melanocyte differentiation (Grønskov et al., 2013; Wei et al., 2013).

Of note, among these different forms of OCA, OCA2 is sometimes associated with Prader–Willi syndrome (PWS) or Angelman syndrome (AS) because the *p* gene is localized in the distal part of the PWS/AS region (Fridman et al., 2003). PWS is a neurogenetic disorder, characterized by obesity, short stature, muscular weakness, intellectual deficiencies, and deviant social behavior (Cassidy et al., 2012; JiangLev-Lehman et al., 1999). Approximately 70% of cases occur when part of the father’s chromosome 15 is deleted. In another 25% of cases, the person has two copies of chromosome 15 from their mother and none from their father. AS is a monogenic neurological disorder characterized by ataxia, intellectual disability, speech impairment, sleep disorders, and seizures. AS occurs due to lack of function in a part of the chromosome 15 inherited from a person’s mother. Most of the time, it is due to a deletion or mutation of the *UBE3A* gene on that chromosome (Bailus and Segal, 2014). PWS and AS were the first recognized human genomic imprinting disorders. Both syndromes are caused by several different genetic alterations in the chromosome region 15q11.2-q13. However, deletion-related PWS and AS cases do not represent a genetically homogeneous group, as they are composed of two main groups: Class I, with breakpoints at BP1 (proximal) and BP3 (distal), and Class II, with breakpoints at BP2 (proximal) and BP3 (distal) (Faundes et al., 2014). The *p* gene is not imprinted, and both alleles are expressed. PWS and AS patients with typical deletions are thus hemizygous for the *p* gene. It is also well-established that PWS and AS deletion patients usually show hypopigmentation of the skin and hair and *p* causes this hypopigmentation as well (Clayton-Smith, 1993; Spritz et al., 1997), although the mechanism has not yet been established.

Here, we reported two OCA2 patients with PWS/AS. The former was OCA2 with PWS due to a novel, atypical deletion of approximately 3.8 Mb in the paternally inherited chromosome 15 and a pathogenic mutation, c.1327G>A (Val443Ile), in the *p* gene of the maternal chromosome. The latter was OCA2 with AS due to a typical deletion in the maternally inherited chromosome 15q11-q13 and a novel mutation, c.1514T>C (Phe505Ser), in the *p* gene of the paternal chromosome. The present study provided valuable information for better evaluation of patients with albinism and more efficient patient identification.

## Subjects and methods

### Patients

**FIGURE 1:**
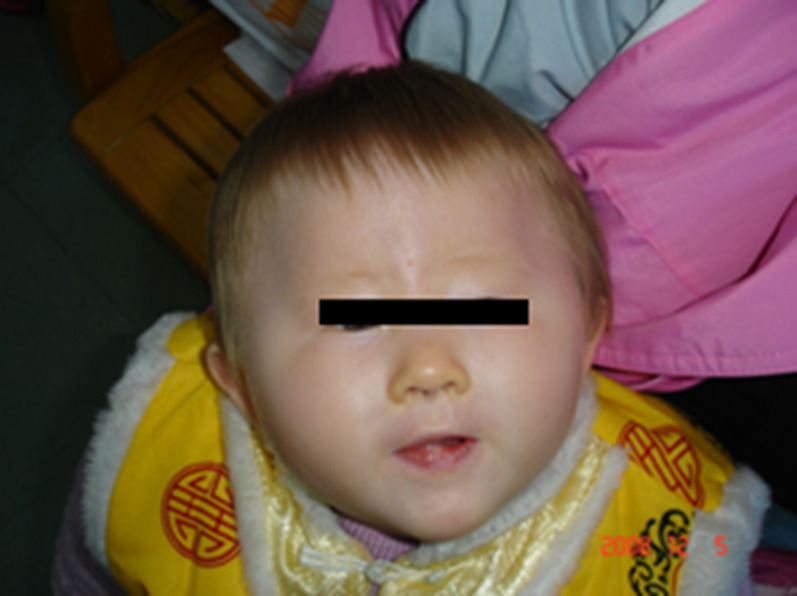
DNA sequencing results of family 1. Proband 1 carried homozygous mutation c.1327G>A in the *p* gene; her father was wild-type (c.1327G); her mother carried heterozygous mutation c.1327G > A/G; the fetus carried heterozygous mutation c.1327G>A/G.

Patient 1 (Proband 1) is a 13-month-old girl born at term after a normal pregnancy. She had normal birth weight, length, and head circumference but had light yellow hair, gray eyes, and milky skin with hypotonia (Figure 1). Her mother (23 years old) and father (27 years old) had normal phenotypes.

**FIGURE 2:**
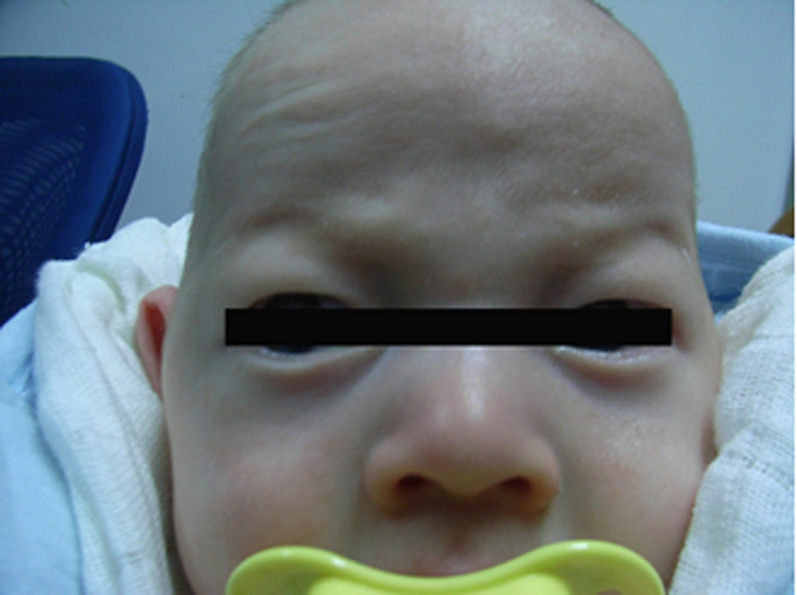
DNA sequencing results of family 2. Proband 2 carried homozygous mutation c.1514T>C in the *p* gene; his father carried heterozygous mutation c.1514T>C/T; his mother was wild-type (c.1514T); his older brother was wild-type (c.1514T).

Patient 2 (Proband 2) is a 50-day-old male newborn with an albinism phenotype; no other obviously abnormal phenotypes were found (Figure 2). His parents and older brother had normal phenotypes.

### Mutation screening of OCA genes

After obtaining written informed consent from all participating individuals, peripheral blood samples and an amniotic fluid sample were collected from the two families. Genomic DNA was extracted using a DNA extraction kit (TIANamp Blood Genomic DNA Purification Kit; Tiangen Biotech, Beijing, China). The coding region and the exon–intron boundaries of *TYR*, *p*, *TYRP1*, and *SLC45A2* were amplified by PCR. Mutation screening was performed in the two families using direct DNA sequencing.

### Haplotype analysis

**FIGURE 3:**
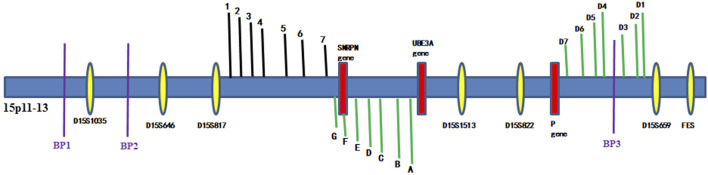
Haplotype schematic map of the members of family 1. The arrow indicates proband 1. She was affected by OCA2 with PWS due to an atypical deletion (chr15: 22330347–26089649, approximately 3.8 Mb) on paternal chromosome 15 and a point mutation c.1327G>A (Val443Ile) in the *p* gene of maternal chromosome 15.

Linkage analysis was performed because the mutations found in the *p* gene violated the laws of classical Mendelian heredity, which suggested that it was likely to be associated with PWS or AS. The fine mapping sequences were obtained from the Human Genome Database (GDB). Haplotype analysis was performed with six microsatellite markers (STRs): D15S646, D15S817, D15S1513, D15S822, D15S659, and D15SFES (as shown in Table 1). To further determine the extent of the deletion, we designed primers 1–7 in the D15S817–D15S1513 direction, the primers A–H in the D15S1513–D15S817 direction, and primers D1–D7 in the D15S659–D15S822 direction on 15q11-q13. The information of these primers is shown in Table 2. Subsequently, primer-walking sequencing combined with SNP linkage analysis was performed to determine the deletion ranges. Prenatal genetic testing was carried out by DNA sequencing and haplotype analysis. Physical sketch maps of the *p* gene, STRs, and primers (1–7, A–D, D1–D7) as well as the typical breakpoints (BP1, BP2, and BP3) on the chromosome region 15q11.2-q13 are shown in Figure 3.

**TABLE 1:** Primers for the STRs.

Primer	Location	STR	Sequence 5′-3′	
D15S646	15q11.2	TAT	F: 5′AGGACAGGAGAAAGGAATTAGGAT3′ R: 5′GCTAGATGACGGGTTAGTGGGT3′	
D15S817	15q11.2	GATA	F: 5′GAACCGTTCATACTACCAAAG 3′ R: 5′TACTGAGGGTTTAACCAAGG 3′	
D15S1513	15q12	GATA	F: 5′ACATCCTCCACGTACGAATAATA 3′ R: 5′CAGGATATGTTTTTTGGGGAT 3′	
D15S822	15q11.2	TCTA	F: 5′GTGTGAAGTGACAGAAGAGAGCA 3′ R: 5′TCCTATTGAGAGTCCATTGAGATT 3′	
D15S659	15q11.2	TATC	F: 5′TGGTAGTCTGGACACCTATTGC 3′ R: 5′AGGCAGTAATGGTTAGTGGAGAA 3′	
FES	15q25.1	TAAA	F: 5′CCCCATCTCTACTGAAAAAGCAA 3′ R: 5′GGGTCACTCTGGGGATTTGG3′	

**TABLE 2:** Primers for the SNPs.

Primer	Location	Sequence 5′-3′
1	chr15: 22273981-22276481	F: 5′TAT​CGC​TTT​TCT​TTT​ACT​GAC 3′
		R: 5′TCC​TTC​CCT​CAA​CTA​CTC​C 3′
2	chr15: 22297213-22297826	F: 5′CAT​CAC​CCC​TCT​GGT​TGT​CAT 3′
		R: 5′TCC​AGA​CCC​AGG​GAC​TAT​CAT 3′
3	chr15: 22312426-22313016	F: 5′ACT​TAC​TAT​AAG​CGA​CTA​CCA​TT 3′
		R: 5′TTT​CTA​GTG​TGA​TAT​TTG​GAG​AC 3′
4	chr15: 22313906-22314256	F: 5′ACT​CCT​TAA​CAT​ATT​GGG​GTG​A 3′
		R: 5′CCT​GGG​AGT​CCA​GAG​AGA​GA 3′
5	chr15: 22322315-22323034	F: 5′CAA​ATA​CGG​AAT​GTG​AGG​TCC​TG 3′
		R: 5′CAC​AAG​ACA​AAT​GCA​CAA​TCA​ACT 3′
6	chr15: 22326235-22326805	F: 5′TTC​AAA​TTC​CTA​GCC​TCA​AGC​AG 3′
		R: 5′AGC​TGT​GCC​CTC​TGA​CTA​CAT​TG 3′
7	chr15: 22329848-22330347	F: 5′AAG​GGT​ATG​GTT​CTG​TGT​GTA​AG 3′
		R: 5′AGT​TTC​AAG​TTT​ATC​ATA​AAA​CCA 3′
A	chr15: 22790005-22790715	F: 5′CTG​TTG​GGT​GCC​TGG​ATA​GA 3′
		R: 5′ATG​GTT​ATG​AAA​ATG​AGG​TGC​T 3′
B	chr15: 22518350-22519347	F: 5′CAC​AGC​GAA​AGA​AAC​TAT​CAA​T 3′
		R: 5′AGG​TCA​ATA​AAG​AAA​TCA​ATG​G 3′
C	chr15: 22458684-22459484	F:5′GAGCTCATATGCAGAAACTAA 3′
		R:5′TAATACTGGAAAATGACACAAA 3′
D	chr15: 22438713-22439295	F: 5′CTT​TGA​AGA​AAA​TCC​CAG​AGA 3′
		R: 5′CTA​TGT​CTT​GAA​AAA​TTG​CCA 3′
E	chr15: 22435496-22436048	F: 5′GAA​CAG​CAA​ATG​TTC​CTG​CC 3′
		R: 5′GGT​CAC​TCC​CAC​CCT​AAT​AAT​G 3′
F	chr15: 22429425-22429940	F: 5′GCT​CAT​TCC​CTG​TTT​ACT​GTG 3′
		R: 5′ATT​TTT​TGC​TTC​CAG​ACC​TAT 3′
G	chr15: 22330366-22330837	F: 5′AAT​TGT​ACC​TTG​CCA​CTT​TG 3′
		R: 5′CAG​TCA​TAC​CTA​ATC​TCT​TTC​C 3′
D1	chr15: 43844144-43844774	F: 5′TCA​CTG​GAT​GTG​AGC​TGT​CC 3′
		R: 5′GCA​AGT​AGG​CAA​AAA​GAA​GAG 3′
D2	chr15: 39698389-39698897	F: 5′GTA​CTC​CCT​TCC​TGC​TCC​CT 3′
		R: 5′AAG​TGT​AGC​CAC​AAG​AGA​CCA 3′
D3	chr15: 32986240-32986885	F: 5′ATT​TCA​TTG​TTT​TCA​GTC​TTT 3′
		R: 5′TCT​TCC​AGT​TTC​TCT​ACT​TTT​A 3′
D4	chr15: 28835913-28836416	F: 5′GCG​ATC​TCA​GCT​CAC​TAC​AAC 3′
		R: 5′AAC​CTT​GGT​AAA​CTG​AGG​CAA 3′
D5	chr15: 27117598-27118068	F: 5′CCA​GGA​CTC​TGT​AAG​CTC​TAG 3′
		R: 5′TTC​ACC​TAA​TGC​TTC​AGT​ACT​A 3′
D6	chr15: 26153681-26154397	F: 5′GGA​TAG​ATC​CTT​GGA​GAT​GGG​A 3′
		R: 5′CAG​CAG​TCC​TGA​AAT​TGC​ATG​A 3′
D7	chr15: 26089649-26090302	F: 5′CCA​TAA​GGA​AAA​TAT​TAA​AAC​CA 3′
		R: 5′CAG​CAT​CTG​CTT​TTA​TGA​ATA​AC 3′
P13	chr15: 25601391-25947825	F: 5′TCT​CGG​CCC​CCC​TAG​GAC​AT 3′
		R: 5′CTC​AAC​CGC​CCC​CAC​CTT​TT 3′

### Pathogenicity evaluation of the novel mutation

The mutation sites were acquired from the Human Gene Mutation Database (http://www.hamd.cf.ac.uk/), Hermansky–Pudlak Syndrome database (http://liwewilab.genetics.ac.cn/HPSD/), Albinism Database (http://albinismdb.med.umn.edu/) and SNP database (http://www.ncbi. nlm.nih.gov/SNP/), as well as the Ensembl website (http://asia. ensembl.org/index.html) to exclude the possibility of common polymorphisms. In addition, the pathogenicity of mutation sites was also analyzed using web-based computational pathogenicity prediction tools including Align GVGD (http://agvgd.iarc.fr/agvgdinput.php), SIFT (http://sift.jcvi.org/), and PolyPhen-2 (http://genetics.bwh.harvard.edu/pph2/).

## Results

### Mutation screening results

**FIGURE 4:**
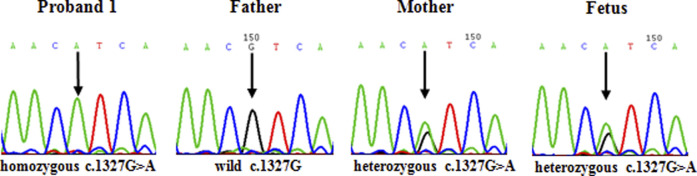
Haplotype schematic map of the core members of family 2. The arrow indicates proband 2. He was diagnosed as OCA2 with AS due to a typical maternal deletion on maternal chromosome 15q11-13 and a novel pathogenic point mutation c.1514T>C (Phe505Ser) in the *p* gene of paternal chromosome 15.

The mother of proband 1 was heterozygous for c.1327G>A (Val443Ile) in exon 13 of the *p* gene, and the father of proband 1 was wild-type (c.1327G) at this site. However, for proband 1, only one pathogenic homozygous mutation, c.1327G>A (Val443Ile), was detected in exon 13 of the *p* gene, violating the laws of classical Mendelian heredity. In prenatal diagnosis, the fetus was a carrier of the heterozygous mutation c.1327G>A in the *p* gene. The sequencing results are shown in Figure 4.

**FIGURE 5:**
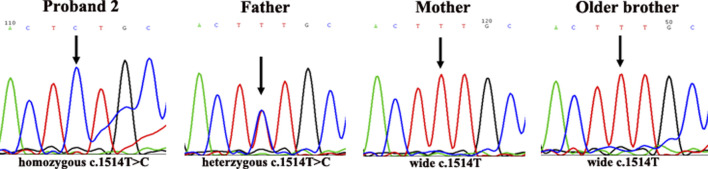
Conservation comparison results of *p*.505F in ten species. This site is highly conserved among ten species.

In the second family, the father of proband 2 was heterozygous for c.1514T>C (Phe505Ser) in exon 15 of the *p* gene, while the mother and older brother of proband 2 were wild-type (c.1514T) at this site. Interestingly, in proband 2, only one novel homozygous mutation, c.1514T>C (Phe505Ser), was detected in exon 15 of the *p* gene (as shown in Figure 5).

### Linkage analysis results

**FIGURE 6:**
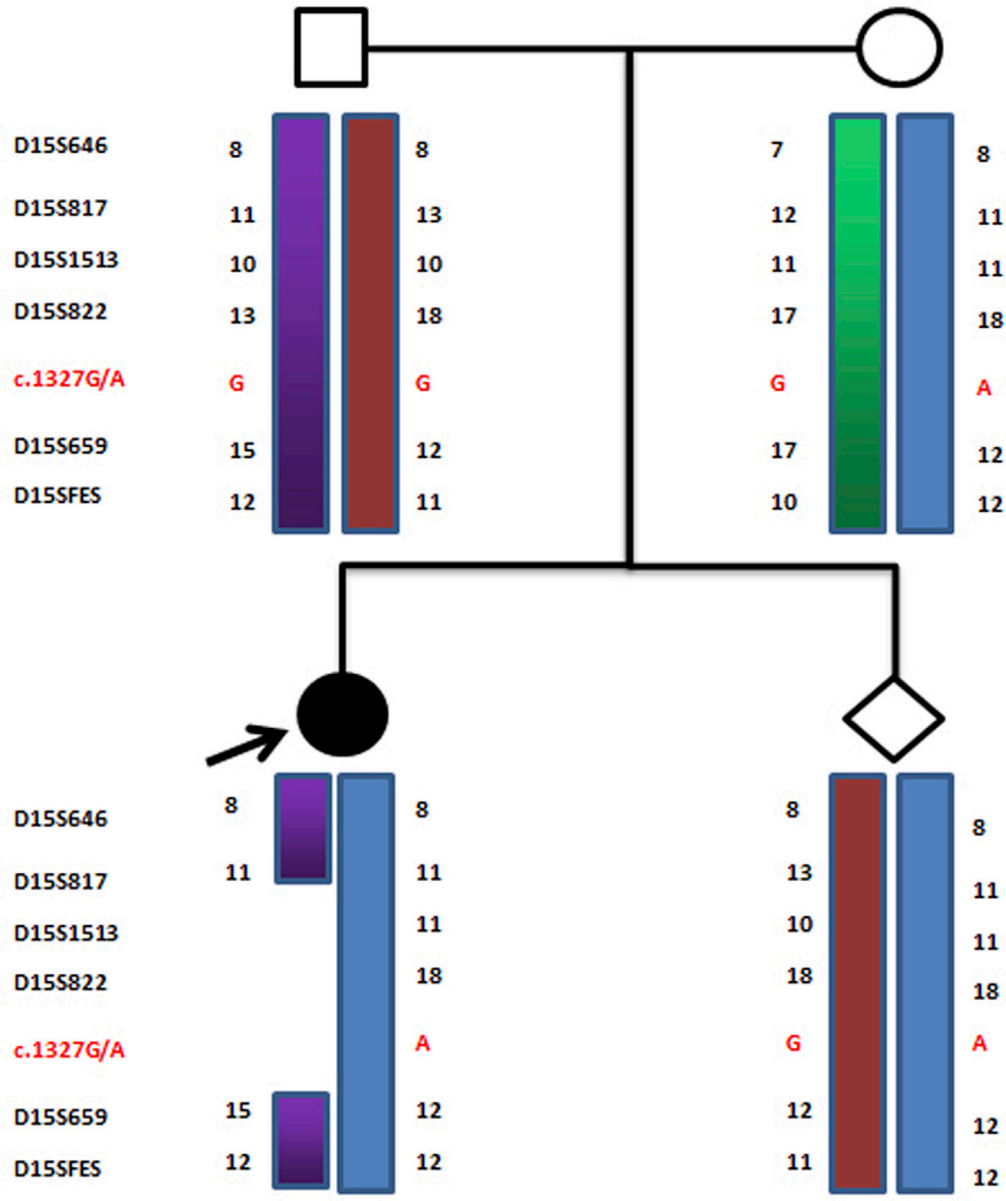
Photograph of proband 1 at 13 months. She was diagnosed as OCA2 with PWS.

Because of these “violations” of the laws of Mendelian heredity, theoretically speaking, three possibilities exist: first, *de novo* mutations occurring during the development of the fertilized zygote; second, uniparental disomy (UPD) of chromosome 15; and third, a deletion mutation in paternal chromosome 15. According to a linkage analysis of family 1, paternal deletions were present at D15S1513 and D15S822, but normal alleles were found at D15S646, D15S817, D15S659, and D15SFES in proband 1. The results of the linkage analysis are shown in Figure 6. Because D15S646, D15S817, D15S1513, and D15S822 are located between breakpoint 2 and breakpoint 3 of the PWS/AS chromosomal region and D15S659 and D15SFES are outside the region, these results suggested that a microdeletion was present on chromosome 15, smaller than that of the internationally reported Class II. Primer-walking sequencing combined with SNP linkage analysis revealed that proband 1 had a microdeletion (chr15: 22330347-26089649, approximately 3.8 Mb) between primers G and D7, which was in line with the distal breakpoint BP3 but significantly below the upstream proximal breakpoint BP2. Therefore, this variation represents a novel deletion found in PWS. In prenatal diagnosis of family 1, linkage analysis confirmed that the fetal chromosome was normal in the PWS region, as shown in Figure 6.

**FIGURE 7:**
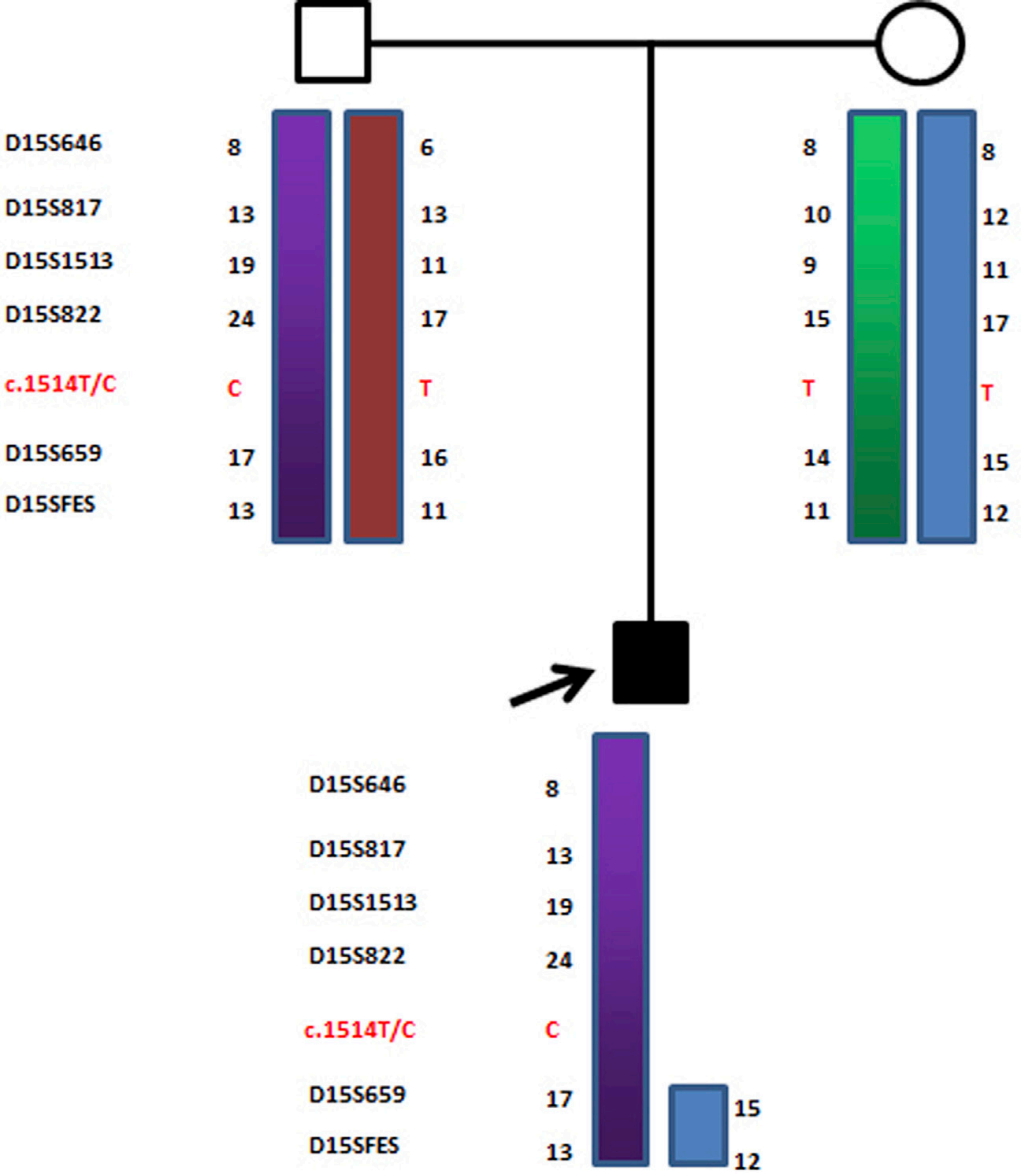
Photograph of proband 2 at 50 days. He was diagnosed as OCA2 with AS.

Similarly, in proband 2, D15S659 and D15SFES were inherited from both parents, but D15S646, D15S817, D15S1513, and D15S822 showed maternal deletion (as shown in Figure 7). STR linkage analysis combined with DNA sequencing revealed that proband 2 suffered from OCA2 with AS.

### Pathogenicity evaluation of the novel mutation

**FIGURE 8:**
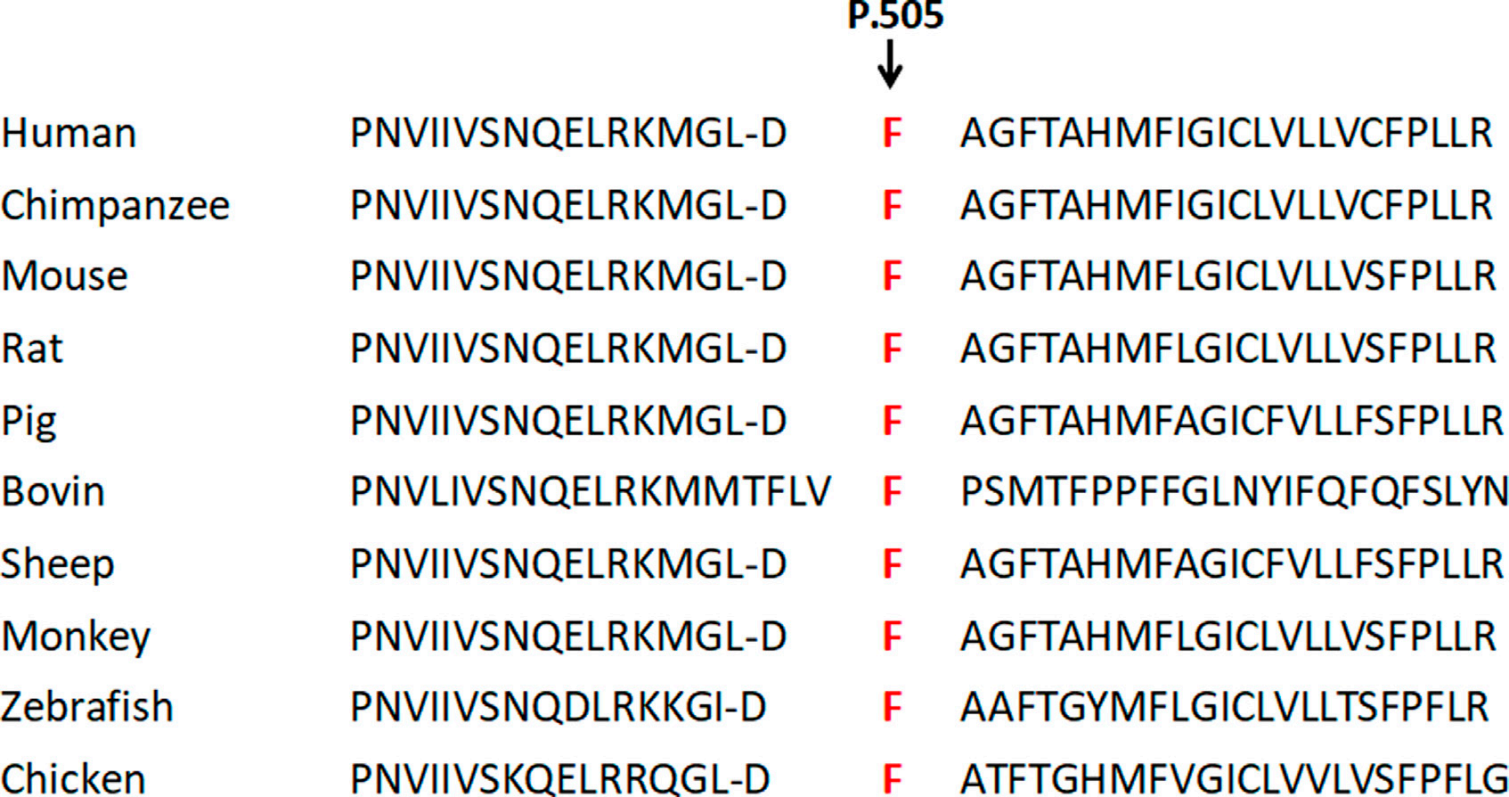
Physical sketch map of the *p* gene, STRs, and primers (1–7, A–D, D1–D7) as well as the breakpoints on the chromosome region 15q11.2-q13. (BP1, breakpoint 1; BP2, breakpoint 2; BP3, breakpoint 3).

After a careful assessment using the Human Gene Mutation Database, Hermansky–Pudlak syndrome Database, Albinism Database and SNP database, as well as the Ensembl website to exclude the possibility of a common polymorphism, a previously potential unreported mutation c.1514T>C (Phe505Ser) on the *p* gene was considered. This site is highly conserved among ten species, i.e., human, mouse, monkey, chimpanzee, pig, cattle, chicken, sheep, rat, and zebrafish, as shown in Figure 8. Web-based computational pathogenicity prediction tools including Align GVGD, SIFT, and PolyPhen-2 indicated that this novel missense mutation is pathogenic.

### Follow-up

Proband 1 showed microcephaly and partial myelinization at 6 months using magnetic resonance imaging (MRI). In addition, her occipitofrontal head circumference (OFC) was at the third percentile at 1 year, and she had hypotonia, unusual hand movements, and developmental delays. Then, she exhibited over-eating, almond-shaped eyes, low ears, narrow eyes, fat, and smaller hands and feet. In addition, her hair became fawn-colored, and she exhibited language barriers and hypoplasia of sexual organs with increasing age. The newborn in family 1 had a normal phenotype at birth.

Proband 2 exhibited developmental retardation, dyskinesia, and language barriers during growth and development, which corresponded to the symptoms of AS. In addition, when proband 2 developed a fever (body temperature exceeding 38.5°C), febrile seizure occurred.

## Discussion

Generally, the clinical recognition of OCA is relatively easy because of the cutaneous hypopigmentation associated with the presence of nystagmus, foveal hypoplasia with little retinal melanin pigment, and reduced visual acuity. However, determining the specific type of OCA still requires diagnostic techniques based on molecular biology. Furthermore, OCA is sometimes associated with PWS/AS because of their similar genomic regions: the *p* gene corresponding to OCA2 is located on chromosome 15q11-q12, within the PWS/AS chromosome region.

In this study, we performed a comprehensive mutational analysis. Finally, the causes of the probands’ conditions were identified. Proband 1 was affected with OCA2 and PWS with an atypical deletion (approximately 3.8 Mb) on paternal chromosome 15 and the pathogenic mutation c.1327G>A (Val443Ile) in the *p* gene of the maternal chromosome. The atypical deletion mutation was smaller than the classical deletion mutation. In addition, we performed prenatal genetic diagnosis for family 1 and found that the fetus was a heterozygous carrier (c.1327G>A/G) with a normal phenotype. Proband 2 was diagnosed as OCA2 with AS due to a typical maternal deletion on chromosome 15 and a novel mutation, c.1514T>C (Phe505Ser), in the *p* gene of the paternal chromosome.

A previous study has shown that the prevalence of OCA2 resulting from the 2.7-kb deletion in exon 7 of the *p* gene is 1 in 1,800 in individuals of African origin (Durham-Pierre et al., 1994). However, the incidence of OCA caused by deletions in chromosome 15 and point mutations in the *p* gene remains unknown in the Chinese population. The current study showed that there existed deletion mutations in the OCA gene that failed to be detected through direct sequencing. Molecular classification of PWS/AS is important because it indicates a risk of recurrence in the siblings of PWS/AS patients. Thus, a precise determination of molecular class in PWS and AS patients is essential for accurate genetic counseling of parents seeking a future pregnancy (Fridman et al., 2003; Cassidy et al., 2012). The recognition of OCA with PWS/AS is as important as early treatment to improve the prognosis of this dismal disease, enhance survival and quality of life, and provide a basis for prenatal diagnosis.

In our study, a new deletion mutation of PWS was found through primer-walking sequencing and SNP analysis of 15q11-13. The discovery of this novel variation enriched the known genetic heterogeneity of PWS in China. As various degrees of hypopigmentation are associated with PWS and AS patients, the study of the *p* gene in a hemizygous state could contribute to the understanding of its effect on human pigmentation during development and potentially disclose the presence of modifier pigmentation gene (s) in the PWS/AS region (Fridman et al., 2003; Spritz et al., 1997).

## Conclusion

In this study, we reported on two OCA families. One was affected by OCA2 with PWS due to an atypical deletion (chr15: 22330347–26089649, approximately 3.8 Mb) on paternal chromosome 15 and a point mutation c.1327G>A (Val443Ile) in the *p* gene of maternal chromosome 15, and we performed prenatal diagnosis for family 1, which indicated that the fetus was a heterozygous carrier (c.1327G>A/G in the *p* gene) with a normal phenotype. Another family was diagnosed as OCA2 with AS due to a typical maternal deletion on maternal chromosome 15q11-13 and a novel pathogenic point mutation c.1514T>C (Phe505Ser) in the *p* gene of paternal chromosome 15. These findings increased the spectrum of clinical conditions associated with *p* gene mutations. Our study indicates the necessity of further investigation for concurrent PWS/AS in patients with developmental delays and albinism.

## Data Availability

The datasets presented in this study can be found in online repositories. The names of the repository/repositories and accession number (s) can be found in the article/Supplementary Material.
